# Gene expression alterations from reversible to irreversible stages during coral metamorphosis

**DOI:** 10.1186/s40851-022-00187-1

**Published:** 2022-01-25

**Authors:** Yuu Ishii, Masayuki Hatta, Ryusaku Deguchi, Masakado Kawata, Shinichiro Maruyama

**Affiliations:** 1 Department of Biology, Miyagi University of Education, Aoba-ku, Sendai, Sendai, Miyagi 980-0845 Japan; 2 Department of Environmental Life Sciences, Graduate School of Life Sciences, Tohoku University, Aoba-ku, Sendai, Miyagi 980-8578 Japan; 3 Graduate School of Humanities and Sciences, Ochanomizu University, Bunkyo-ku, Tokyo, 112-8610 Japan

**Keywords:** *Acropora*, Metamorphosis, RNA-seq, GPCR, Proteolysis

## Abstract

For corals, metamorphosis from planktonic larvae to sedentary polyps is an important life event, as it determines the environment in which they live for a lifetime. Although previous studies on the reef-building coral *Acropora* have clarified a critical time point during metamorphosis when cells are committed to their fates, as defined by an inability to revert back to their previous states as swimming larvae (here referred to as the “point of no return”), the molecular mechanisms of this commitment to a fate remain unclear. To address this issue, we analyzed the transcriptomic changes before and after the point of no return by inducing metamorphosis of *Acropora tenuis* with Hym-248, a metamorphosis-inducing neuropeptide. Gene Ontology and pathway enrichment analysis of the 5893 differentially expressed genes revealed that G protein-coupled receptors (GPCRs) were enriched, including GABA receptor and Frizzled gene subfamilies, which showed characteristic temporal expression patterns. The GPCRs were then classified by comparison with those of *Homo sapiens*, *Nematostella vectensis* and *Platynereis dumerilii*. Classification of the differentially expressed genes into modules based on expression patterns showed that some modules with large fluctuations after the point of no return were biased toward functions such as protein metabolism and transport. This result suggests that in precommitted larvae, different types of GPCR genes function to ensure a proper environment, whereas in committed larvae, intracellular protein transport and proteolysis may cause a loss of the reversibility of metamorphosis as a result of cell differentiation.

**Supplementary Information:**

The online version contains supplementary material available at 10.1186/s40851-022-00187-1.

## Background

Planula larvae of corals determine their positions of attachment while dispersing in ocean currents, which consequently influences the limits of their distribution in the ocean [1]. Various factors, such as sensory system stimulation [2], surface structure [3], and chemical substances [4–7], are involved in the transition from swimming larvae to sessile polyps. When larvae encounter signals that are restricted to small areas of substratum, they cease to swim, rest on the substratum on their aboral end and assume a round shape (Supplementary Fig. 1). If the larvae receive sufficient stimuli from the substratum, they undergo metamorphosis, and stable attachment to the substratum is realized as a result of irreversible cell differentiation processes.

In the genus *Acropora,* which is used as a model for developmental studies of reef-building corals, cues from crustose coralline algae (CCA) covered by their surface biofilms [8–10] have been shown to affect the transition of the planula larvae. Settlement cues from CCA and its surface biofilm are thought to be received by planulae, presumably via sensory neurons [11], and converted into signals to undergo metamorphosis. Alternatively, Hym-248, a GLWamide neuropeptide found in freshwater *Hydra* [12–17], is unique in that, unlike endogenous neuropeptides examined thus far, it can experimentally trigger metamorphosis of acroporids, presumably by mimicking a native internal signaling ligand [12, 15]. As Hym-248 induces metamorphosis without larvae needing to rest on substrata, this exogenous molecule may bypass physiological changes that precede the induction of metamorphosis [18, 19]. Thus, Hym-248-induced metamorphosis is an ideal model system for examining the complex signaling processes and early stages of metamorphosis.

Metamorphosing larvae can revert back to swimming and seek other stopping places even when apparently favorable environmental signals are present (Supplementary Fig. 1), suggesting that sufficient metamorphosis stimuli beyond a ‘threshold’ must be received for the progression of morphogenesis. Similarly, although treating larvae of some coral species (e.g., *Acropora tenuis*) with Hym-248 causes a morphological change from the swimming to the benthic form, a previous study showed that planula larvae returned to the swimming form if Hym-248 was removed within 4 h at 26 °C [14]. This result suggests that the metamorphosis process can be divided into at least two phases: one is a reversible phase where the level of metamorphosis stimuli is below the threshold, and the other is an irreversible phase where the larval cells are committed to certain cell fates and further progress in metamorphosis after sufficient stimuli are received. Here, we call the critical dividing point the “point of no return” (PNR).

Although gene expression profiles from ESTs and RNA-seq have been analyzed in many studies to search for genes involved in planular metamorphosis [18–22], to our knowledge, no studies have focused on irreversible metamorphosis around the PNR. To better understand the process of metamorphosis of the planula, we focused on the early stages of metamorphosis and identified the metabolic pathways potentially involved in determining reversible metamorphosis.

## Materials and methods

### Collection and maintenance of *Acropora* planula larvae

Colonies of *A. tenuis* were collected with permission from Okinawa Prefecture (#31–65) at Sesoko Island (26° 37′41″N, 127°51′38″E, Okinawa, Japan). Corals were kept in tanks with running natural seawater and under partially shaded natural light at Sesoko Marine Station (University of Ryukyus, Okinawa, Japan). After spawning on June 7, 2020, bundles of aposymbiotic gametes from ten colonies were mixed, and the resulting planula larvae were maintained in plastic bowls at approximately 1000 larvae/L in 10 μm filtered natural seawater (FNSW) or artificial sea water (ASW) using Instant Ocean sea salt (Aquarium systems); the water was exchanged daily.

### Induction of metamorphosis and sampling for RNA-seq

At 17 days postfertilization (dpf), larvae were induced to metamorphose in 6-well plates with 200 individuals/3.5 ml ASW supplemented with 16 μM Hym-248 neuropeptide (NH2-EPLPIGLW-CoNH2, synthesized by Scrum Inc., Japan). The timing of metamorphosis induction and the concentration of planula were determined based on previous studies [23, 24], respectively. After the addition of Hym-248, the morphology was observed using a stereomicroscope (OLYMPUS SZX-FOF, OLYMPUS DP 27, Olympus, Japan) at 0 (before treatment), 0.5, 1, 2, 4, 6, and 24 h. Samples for RNA extraction were obtained at 0, 1, and 6 h. For RNA extraction, 20 individuals were soaked in 1.8 ml of RNAlater (Thermo Fisher Scientific, Massachusetts, USA) and stored at − 80 °C until RNA extraction.

### RNA extraction and sequencing

RNA was extracted from one single larva preserved in RNAlater (4 biological replicates for each time point, 0, 1 and 6 h). After blotting to remove excess RNAlater, RNA extraction was performed according to the protocol of the Maxwell® RSC Plant RNA Kit (Promega, Wisconsin, USA). The quality and quantity of RNA were verified using an Agilent RNA 6000 Pico Kit on an Agilent Bioanalyzer (Agilent Technologies, California, USA) and a Nanodrop spectrophotometer (Thermo Fisher Scientific), respectively. We confirmed that the Bioanalyzer RIN values ranged from 6 to 9.6 and that all the total RNA spectra were quite similar and suitable for RNA-seq samples. Ten nanograms of total RNA was used for cDNA amplification with the SMART-Seq® v4 Ultra® Low Input RNA Kit for Sequencing (Takara Bio, Japan). Total cDNA samples from planulae were subjected to library preparation using the NEB Next Ultra RNA Library Prep Kit (New England Biolabs, Ipswich, MA, USA) according to the manufacturer’s protocol (NEB #E7530). These mRNA libraries were sequenced on an Illumina NovaSeq6000 (S2 flow cell) in dual flow cell mode with 150-mer paired-end sequences (Filgen Inc., Japan).

### Transcriptome analysis

A total of 12 libraries (four larva at each of 0, 1 and 6 h) were obtained and filtered using fastp (with options -q 30 -u 30), and paired output reads were used for analysis. The reads from each library were mapped onto the *A. tenuis* genome sequences [25] using STAR 2.7 [26] with the default setting. The read count data, transcripts per million (TPM), were calculated using RSEM [27].

To obtain a visual overview of the effect of Hym-248 treatment time on the global gene expression patterns, principal component analysis (PCA) was performed on the TPM values using the function “prcomp” in R. Genes harboring 5 or more mean counts across samples were included in the PCA. Differentially expressed genes (DEGs) among the three groups were detected by a likelihood ratio test using the R package “edgeR” [28] with the count data as input. To define DEGs or genes showing statistically significant differences in expression among the three groups, a false discovery rate (FDR), or *q*-value, of 0.01 was used as a cutoff.

### GO term and KEGG pathway enrichment analysis

InterProScan 5.48 [29] was used to annotate *A. tenuis* protein sequences enriched with Gene Ontology (GO) terms, resulting in 14,482 genes linked to GO terms. GO term enrichment analysis was performed using the “goseq” package in R, which can compensate for the gene length bias and calculate *p* values for each GO category using the Wallenius approximation to test over- and underrepresentation among the differentially expressed genes [30]. KAAS [31] was used to annotate the *A. tenuis* protein sequences for Kyoto Encyclopedia of Genes and Genomes (KEGG) pathway enrichment analysis, resulting in 11,283 genes possessing KO annotation. The resulting KO annotations were mapped to the KEGG pathway using the KEGG mapper [32]. Enrichment analysis of the KEGG pathway was performed using WebGestalt [33] based on the obtained KO annotation-KEGG pathway information, with the option ‘weighted set cover’ for redundancy reduction.

### Identification and characterization of *A. tenuis* GPCRs

To search for the GPCR homologs in *A. tenuis*, a domain search of Pfam [34] was carried out using InterProScan 5.48 [29] for all peptide sequences of *A. tenuis*, and the genes associated with 45 types of Pfam accessions belonging to the ‘Family A G protein-coupled receptor-like superfamily GPCR_A (CL0192)’ were extracted. The obtained TPM values were normalized by the ‘trimmed mean of M values’ (TMM) method using the R package “edgeR”. The z scores of each normalized TPM value for each gene were calculated in R (Supplementary material 1). The heatmap of the z scores was created using the function “heatmap” in R.

For clustering, the protein sequences of the GPCR homologs of *A. tenuis* and the protein sequences belonging to the ‘Family A G protein-coupled receptor-like superfamily’ of *Homo sapiens*, *Nematostella vectensis*, and *Platynereis dumerilii* (*Platynereis* data contained only Family 1 and “others”) were analyzed in CLANS [35] (Supplementary Data 1, 2, 3, 4). All *Homo sapiens* GPCR protein sequences associated with each Pfam accession (reviewed in (Swiss-Prot), using a manually annotated filter) from the UniProt database were used. *Nematostella* GPCR protein sequences were identified based on the Pfam domain annotations found in a previous study [36]. Based on the GPCR protein sequences found in a previous study [37], *Platynereis* GPCR protein sequences were classified into each family by the Pfam domain search in InterProScan 5.48 [29]. CLANS input files were created using the MPI Bioinformatics Toolkit [38] (Scoring Matrix BLOSUM 62, E-value is 1e − 40). CLANS was performed approximately 10,000 times and was drawn with Cluster in 2D.

### Analysis of gene modules with time-dependent expression patterns

The TPM data normalized by the TMM method were also used for weighted gene coexpression network analysis (WGCNA [39]) to identify coregulated groups of genes (modules). The signed adjacency matrix was calculated using a soft threshold power of 12 and a minimum module size of 30. Modules were characterized by GO enrichment performed using the “goseq” package in R [30]. The z scores calculated from the normalized TPM values for each gene were used to draw line charts for each module using the R package “ggplot2” [40]. The hub gene of each module was searched using the ChooseTopHubInEachModule of WGCNA. PANTHER annotations output by InterProScan 5.48 [29] were used for analysis.

## Results

### Morphological and gene expression changes during metamorphosis after the addition of Hym-248

**Fig. 1:**
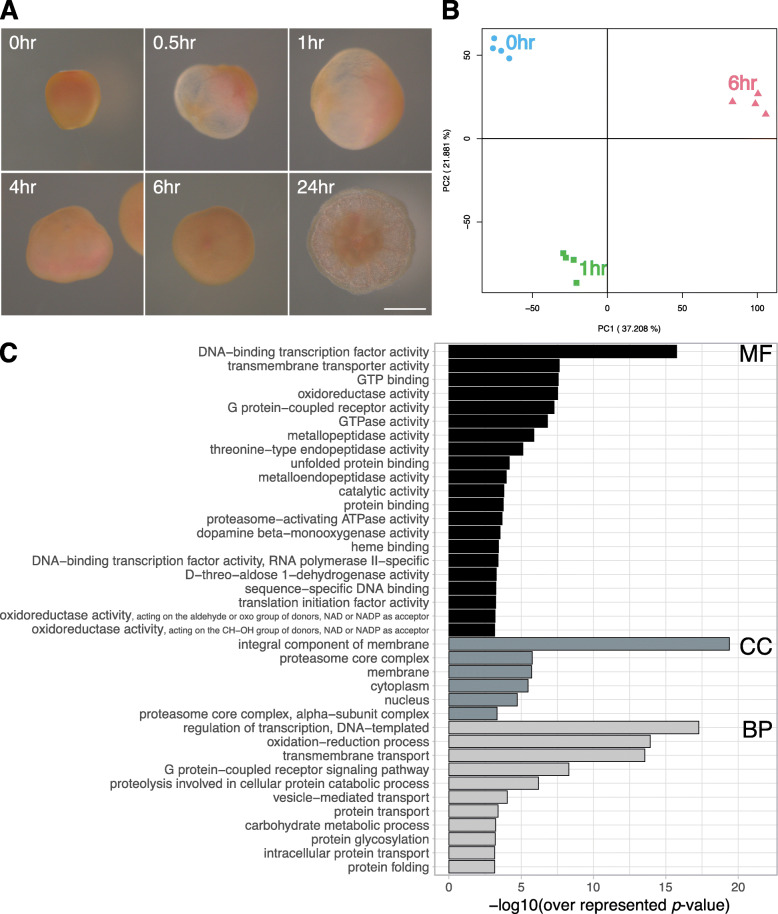
Global pattern of gene expression in samples used for RNA-seq analysis. **A** Morphological changes in *Acropora tenuis* after the addition of Hym-248. The samples at 0 (i.e., before the addition of Hym-248) h, 1 h, and 6 h were subjected to RNA-seq analysis. Scale bar = 100 μm. **B** PCA of gene expression patterns of each RNA-seq sample. **C** GO enrichment analysis of DEGs based on three categories: biological process (BP), cellular component (CC), and molecular function (MF)

To confirm the effect of Hym-248 on metamorphosis, morphological changes in *A. tenuis* planula larvae were characterized after the addition of Hym-248 (Fig. 1A). Morphological changes were detectable within 30 min: larvae became rounded and then gradually flattened. At 6 h, they became almost flat, and after 24 h, they showed the form of a juvenile polyp with early skeleton formation. Such morphological changes were in good agreement with those observed in previous studies [41, 42]. After Hym-248 treatment, sampling was carried out at 0 h (before treatment), 1 h (before PNR) and 6 h (after PNR) to obtain transcriptomic data by RNA-seq.

In this study, RNA-seq produced 536,582,100 reads from 12 samples. The total number of mapped reads of the quadruplicates to the reference sequence was 85,433,460 reads for 0 h (pretreated), 74,442,951 for 1 h, and 101,027,843 for 6 h of treatment with Hym-248. The count values of the genes in the transcriptomic dataset were obtained under three conditions (0, 1 and 6 h). Global gene expression patterns were visualized by PCA, and groupwise separation was confirmed (Fig. 1B).

Here, 5893 genes were identified as candidate DEGs related to the metamorphosis process as their expression levels fluctuated significantly among the three groups. GO and KEGG pathway enrichment analyses were performed. GO enrichment analysis showed that terms associated with GPCRs were enriched in both ‘biological process (BP)’ and ‘molecular function (MF)’ categories (BP, G protein-coupled receptor signaling pathway; MF, G protein-coupled receptor activity) (Fig. 1C). In the KEGG pathway analysis, four pathways, including neuroactive ligand–receptor interactions, were enriched (Table 1). These results suggest that the expression of GPCRs, which are involved in neuronal activity, is significantly altered in the early stages of metamorphosis in *A. tenuis*.

**Table 1:** Results of KEGG pathway analysis. All genes: Number of *A. tenuis* genes in the pathway. DEGs: Number of DEGs in the pathway

KEGG pathway	Description	All genes	DEGs	*p* value	FDR
map 04080	Neuroactive ligand–receptor interaction	59	15	5.87E-07	1.1E-05
map 04723	Retrograde endocannabinoid signaling	15	7	1.4E-05	9.1E-05
map 00830	Retinol metabolism	6	3	0.00481	0.02286
map 00603	Glycosphingolipid biosynthesis - globo and isoglobo series	8	3	0.01227	0.03329

### Expression pattern changes of *A. tenuis* GPCR genes during early metamorphosis

**Fig. 2:**
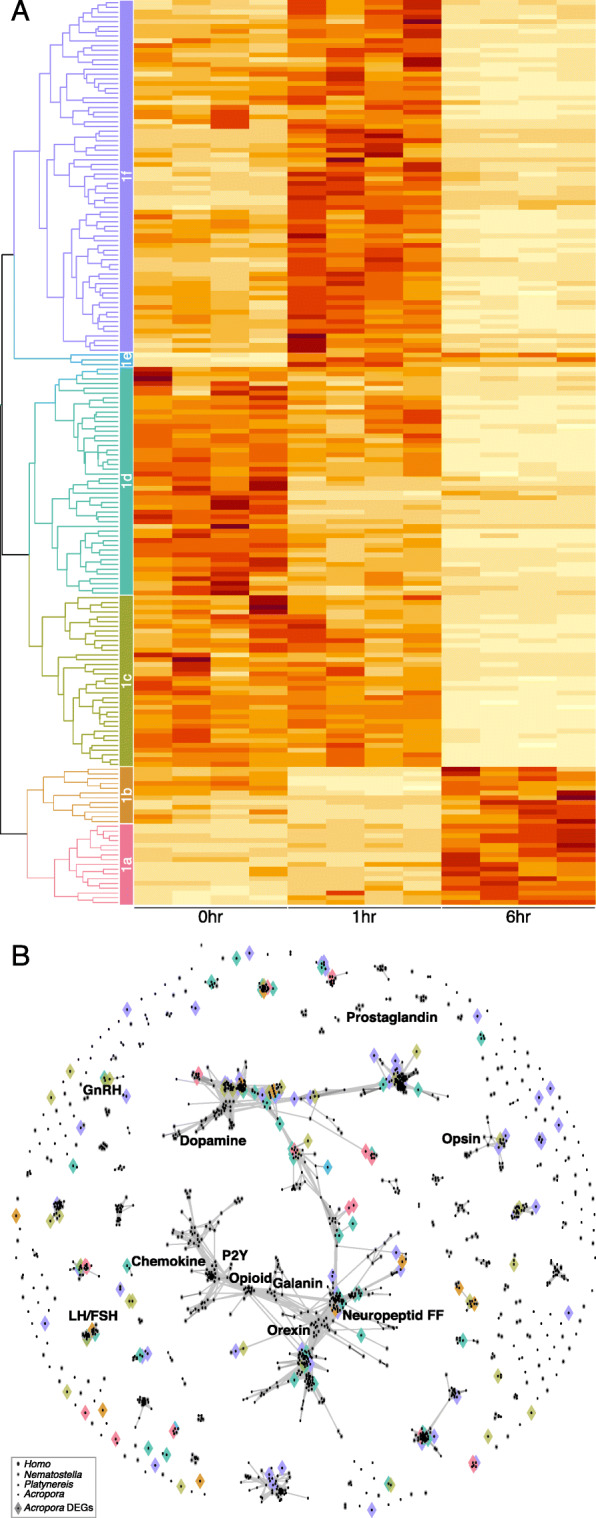
DEGs in GPCR Family 1 of *Acropora tenuis*. **A** Heatmap of gene expression (z score). DEGs were divided into six groups (1a to 1f) according to their expression patterns. **B** Cluster analysis with *Homo sapiens* ligand–receptor annotations. Rhombus indicates DEGs of *A. tenuis*. Rhombus colors correspond to the six DEG groups in Fig. 2A

**Fig. 3:**
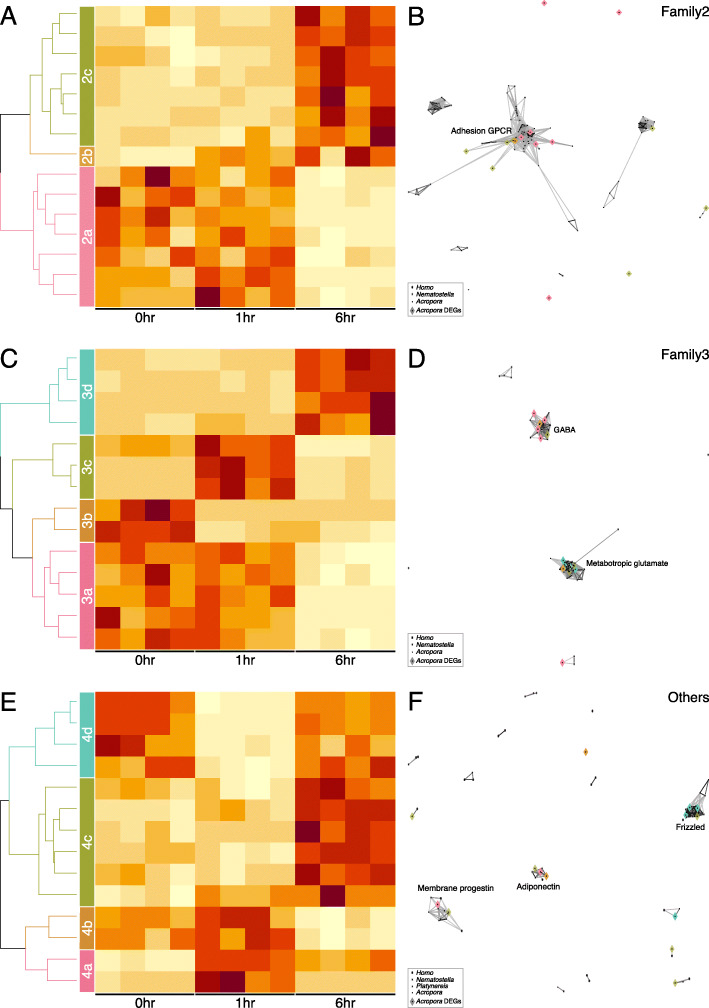
DEGs in GPCR Families 2, 3, and others of *Acropora tenuis*. **A**, **C**, **E**. Heatmap of the gene expression (z score) of Families 2 (**A**), 3 (**C**), and others (**E**). The DEGs were divided into groups according to their expression patterns. **B**, **D**, **F**. CLANS cluster analysis of Families 2 (**B**), 3 (**D**), and others (**F**) with *Homo sapiens* ligand–receptor annotations. Rhombus colors correspond to the DEG groups in Families 2 (**A**), 3 (**C**), and others (**E**)

To further characterize the GPCR genes involved in early metamorphosis, all homologs were identified from the *A. tenuis* genome and classified into Families 1, 2, 3, and “others” based on the Pfam domain definitions. Approximately 20 to 45% of the genes in each family (approximately 25% of the total genes) were identified as DEGs without any apparent familywise bias (Table 2). To visualize the patterns in each GPCR family, heatmaps using z scores of gene expression levels were created, and subfamily level groups were defined based on the expression patterns (Fig. 2A, 3A, C, E). Most GPCRs are orphan receptors for which no ligand has been identified in cnidarians. Therefore, to predict the functions of *A. tenuis* GPCRs from the expression pattern, the GPCR genes were clustered by CLANS, with the *H. sapiens* GPCR sequences used as references. The results showed that although some genes were homologous, there were many *A. tenuis* GPCRs with low homology to *H. sapiens* GPCRs (Fig. 2B, Fig. 3B, D, F).

**Table 2:** The composition of *Acropora tenuis* GPCR families. DEGs were detected by multigroup comparison using 0, 1, and 6 h samples

GPCR family	All genes	DEGs	DEG%
Family 1	802	190	23.69
Family 2	78	15	19.23
Family 3	43	14	32.56
Others	31	14	45.16

DEGs classified as Family 1 were divided into six groups, 1a-1f, based on their expression patterns (Fig. 2A). From the CLANS results, members in each group of Family 1 were distributed throughout the homologous clusters, and no biased GPCR functions were predicted for each group (Fig. 2B). DEGs classified into Family 2 were divided into three groups (2a, 2b, and 2c) based on their expression patterns (Fig. 3A). Regardless of their expression pattern, some members showed high homology to human adhesion GPCRs, but others showed no homology to any human GPCRs (Fig. 3B). DEGs classified as Family 3 were divided into four groups, 3a-3d, based on their expression patterns (Fig. 3C). Group members with increased expression at 1 h or 6 h (groups 3c and 3d) were biased toward metabotropic glutamate receptors (Fig. 3D). In contrast, group 3a, with the highest expression level at 0 and 1 h, was biased toward a cluster of GPCRs acting as gamma aminobutyric acid (GABA) receptors. DEGs classified as “others” were divided into four groups, 4a-4d, based on their expression patterns (Fig. 3E). Group members highly homologous to proteins classified as Frizzled belonged to group 4d; their expression level decreased 1 h after treatment with Hym-248 and recovered at 6 h (Fig. 3F).

### Characteristics of gene modules involved in the early phase of *A. tenuis* metamorphosis

**Fig. 4:**
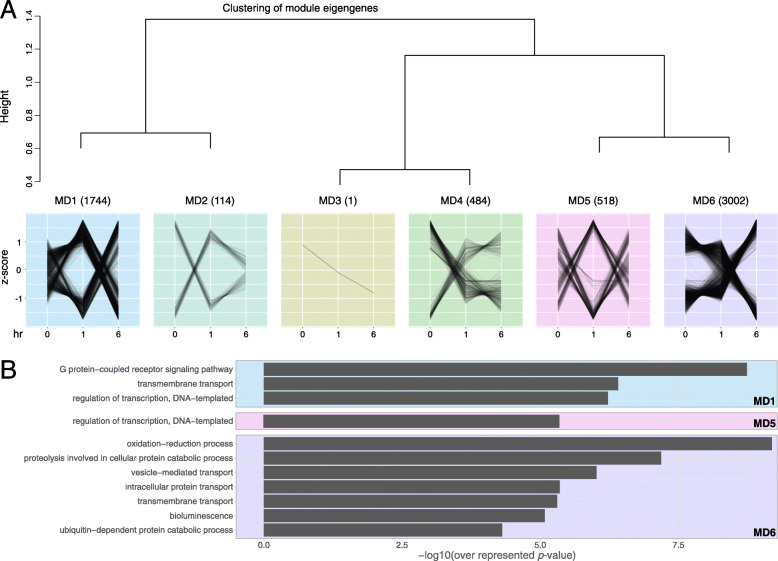
Modular analysis of the expression patterns of the DEGs by WGCNA in the three-group comparison. **A** The dendrogram of the modules and the expression pattern of the genes in each module. The number in parentheses indicates the number of genes clustered into each module. The average values of the z scores of gene expression levels over time are shown by a line plot. **B** GO enrichment analysis for each module. Only BP categories are shown. MD2, 3, and 4 have no enriched terms

To further characterize the gene expression of all DEGs, we conducted WGCNA to classify them into six modules (MD1–6) (Fig. 4A). In addition, the function of each module was characterized by GO analysis (Fig. 4B, Supplementary Tables 1, 2, 3), and hub genes for each module were detected (Table 3). In modules MD2, 3 and 4, no enriched GO terms were detected. In module MD1, GO terms relevant to transcriptional regulation, GPCRs, and transmembrane transport were enriched (Supplementary Table 1). Many of the MD1 genes (1049 out of 1744 genes) showed the highest expression at 1 h. In module MD5, in which the gene expression level showed a large change at 1 h after the addition of Hym-248 and then recovered, the term for transcriptional regulation was enriched, but potential downstream pathways were not found in the enrichment analyses. In module MD6, GO terms associated with redox reactions, protein metabolism (proteolysis, ubiquitin-dependent protein catabolic process, vesicle−mediated transport, and protein transport) and bioluminescence were enriched. In module MD6, eleven genes associated with the GO term ‘bioluminescence’ were all annotated as ‘green fluorescent protein’ by the Pfam domain search analysis. Most of the MD6 genes were expressed at low levels at 0 h and increased at 6 h (6 h z score > 0 in 1812 genes, < 0 in 1190 genes), and the hub gene of this module was RasGAP-like protein 1.

**Table 3:** Hub genes of each module detected by WGCNA

Module	*A. tenuis* gene	Signature accession	Signature description
MD1	aten_0.1.m1.21136.m1	PTHR23051	SOLUTE CARRIER FAMILY 35, MEMBER F5
MD2	aten_0.1.m1.21004.m1	PTHR16243	BTG3-ASSOCIATED NUCLEAR PROTEIN BANP
MD4	aten_0.1.m1.10442.m1	PTHR12981	ZINC FINGER PROTEIN-LIKE 1
MD5	aten_0.1.m1.1137.m1	PTHR13154	POLYADENYLATE-BINDING PROTEIN-INTERACTING PROTEIN 2
MD6	aten_0.1.m1.14993.m1	PTHR10194	RAS GTPASE-ACTIVATING PROTEINS

## Discussion

It is critical to delineate the coral metamorphosis process at the molecular level to further understand how larval dispersal can be regulated, which has a high impact on the diversity and stability of coral reef ecosystems in tropical and subtropical oceans. Previous studies have identified many metamorphosis-related candidate genes by analyzing changes in gene expression at many developmental stages [20] before and after metamorphosis [18, 19] and focusing on metamorphosis competence [21]. In this study, by analyzing the gene expression changes at a fine time resolution before and after the PNR, candidate molecular mechanisms involved in the reversibility of metamorphosis were identified. These results suggest that alterations in environmental signal perception through GPCRs and proteolytic regulation of cellular components can lead to drastic changes that make the metamorphosis process irreversible. These results also show that the gene expression of green fluorescence proteins may be under strict control after the PNR, implying a link to understudied physiological and ecological roles of fluorescence in *A. tenuis* at early developmental stages [43, 44].

### *A. tenuis* differentially regulates GPCR transcripts during early metamorphosis

In this study, a group of genes (GPCRs) were found to have altered gene expression patterns at the early stage of metamorphosis, and the GPCRs with altered expression accounted for approximately 25% of the GPCR homologs of *A. tenuis*. Previous studies have suggested that GPCRs are involved in the metamorphosis of planula larvae in *A. millepora* [21]. This study suggests that the involvement of GPCRs in metamorphosis is conserved in the genus *Acropora* and provides the first comprehensive analysis of the expression patterns of GPCRs at the gene family and subfamily levels.

GABA receptors and Frizzled GPCRs showed dynamic expression patterns in this study. The expression level of the GABA receptor decreased within 1 h after the addition of Hym-248 (Fig. 3C, D). It has been shown that the addition of GABA_B_ antagonists to seawater does not significantly affect metamorphosis itself [21], and GABA receptors are expected to play a role in searching for a substratum before metamorphosis [18] in *A. millepora*. These results suggest that GABA receptors may be involved in the search behavior and that this ability is lost as metamorphosis proceeds because the search behavior of the planula is no longer necessarily activated. Since GABA_B_ receptors are known to stimulate cell differentiation [45], some GABA receptors may be activated at a later developmental stage.

The expression of Frizzled was suppressed 1 h after the addition of Hym-248 (Fig. 3E, F). Frizzled is a receptor involved in the reception of Wnt signaling, and binding of Wnt to Frizzled initiates transcriptional regulation via β-catenin. In *Hydra* and *Hydractinia*, Wnt is expressed in the oral tip and functions as a head organizer [46, 47]. In corals, Wnt may also drive the differentiation of the oral part of the developing polyp during metamorphosis, and changes in Frizzled expression may affect this process. In addition, Ras GTPase Activating Protein (RasGAP)-like protein 1 was detected as a hub gene of MD6, as classified by WGCNA (Table 3), suggesting that Frizzled-Wnt signaling may be mediated by RasGAP after the PNR.

### Protein-level metabolism is involved in the regulation of metamorphosis

**Fig. 5:**
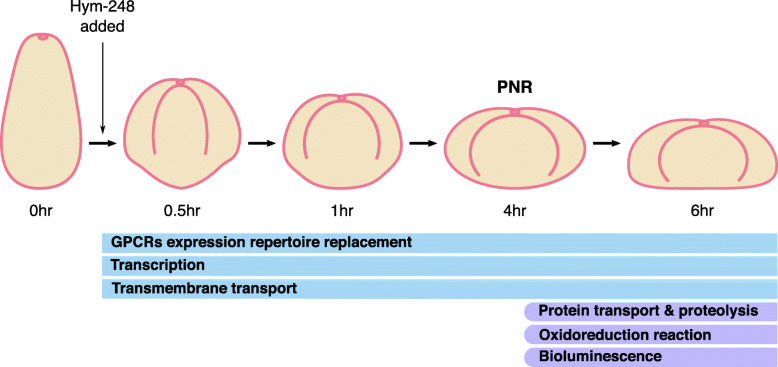
Metabolic pathways expected to be important in the early metamorphosis induced by Hym-248. A model illustrating that in early metamorphosis (between 0 and 6 h; regardless of the PNR) in *A. tenuis*, GPCR expression repertoire replacement, transcription, and transmembrane transport play important roles. After the PNR, the onset of protein transport and proteolysis functions become more relevant to the loss of metamorphosis reversibility. The colors of bands (blue and purple) correspond to the colors of the modules (MD1 and MD6, respectively) where the relevant GO terms are enriched, as shown in Fig. 4

Network analyses identified the gene modules responsible for metabolic pathways potentially involved in the reversibility of metamorphosis (Fig. 5). The results revealed that some metabolic pathways were important throughout the early metamorphosis process, while others made a switch in regulation around the PNR. Transcriptional regulation and receptor signaling were identified as functional characteristics of the precommitted phase, but no specific downstream functions were predicted (see MD1 and, 5 in Fig. 4). In addition to transcriptional regulation, the committed phase after the PNR was characterized by GO terms related to the ubiquitin–proteasome system associated with the genes encoding 26S proteasome and ubiquitin-related enzyme subunits (MD6 in Fig. 4, Supplementary Table 3). This result is consistent with the fact that metamorphosis becomes irreversible after the PNR: compared to the transcription-level regulation detected before the PNR, it would be far more difficult to restore a system to the original form after it has been modified or degraded by proteolytic regulation.

Regulation of proteolysis is important in bacterial development [48] and may play an important role in the progression of coral metamorphosis. During the metamorphosis of *Acropora*, tissue differentiation proceeds rapidly (e.g., formation of the solid, single-layer endoderm and oral organs, including tentacles, mesenteries, and pharynx) [42]. Apoptosis and cell division are known to occur actively after the PNR in the reef building coral *Stylophora* [49]. This result suggests that active tissue reconstitution, including the degradation of existing tissues and the differentiation of new tissues, is preceded by the regulation of the expression of genes related to protein degradation. Although the targets of proteasomal degradation remain unclear, proteolytic regulation may be involved in the downstream differentiation cascades, leading to a complete loss of reversible metamorphosis. Genetic engineering of coral larvae [50] to suppress specific protein degradation processes is an ideal platform to substantiate this hypothesis.

## Conclusions

This study identified key metabolic pathways in the initial stages, especially before and after the PNR, of the metamorphosis of the reef-building coral *A. tenuis*. Comprehensive gene expression pattern analysis suggested that GPCRs with various functions can play important roles in the different phases of coral metamorphosis. In addition, modular analysis of the gene expression fluctuations identified molecular mechanisms associated with the loss of the reversibility of metamorphosis, indicating the onset of proteolytic regulation in the early phases of metamorphosis.

## Supplementary Information

**Additional file 1: Supplementary Fig. 1.** Planula larvae returning to the motile form after resting on the substrata. *Acropora tenuis* planula larvae were released on rubble covered by CCA. Pictures taken 6, 7, and 8 h after release are shown (A, B, and C, respectively). Dotted lines indicate the positions of the larvae at 6 h.

**Additional file 2: Supplementary Table 1.** Enriched GO terms and genes in MD1. **Supplementary Table 2.** Enriched GO terms and genes in MD5. **Supplementary Table 3.** Enriched GO terms and genes in MD6.

**Additional file 3:** **Supplementary data 1.** Input data of CLANS (GPCR Family 1).

**Additional file 4:** **Supplementary data 2.** Input data of CLANS (GPCR Family 2).

**Additional file 5:** **Supplementary data 3.** Input data of CLANS (GPCR Family 3).

**Additional file 6:** **Supplementary data 4.** Input data of CLANS (GPCR others).

**Additional file 7:** **Supplementary material 1.** R script for calculating z scores.

## Data Availability

The dataset supporting the results of this article is included within the article and its supplemental material files. The raw *A. tenuis* RNA-seq data were submitted to DDBJ/EMBL-EBI/GenBank under the BioProject accession number PRJDB12283.

## Abbreviations

ASW: Artificial sea water
BP: Biological process
CC: Cellular component
CCA: Crustose coralline algae
DEGs: Differentially expressed genes
dpf: Days post-fertilization
FDR: False discovery rate
FNSW: Filtered natural seawater
GABA: Gamma aminobutyric acid
GO: Gene Ontology
GPCR: G protein-coupled receptors
KEGG: Kyoto Encyclopedia of Genes and Genomes
KO: KEGG Orthology
MF: Molecular function
PCA: Principal component analysis
PNR: Point of no return
TPM: Transcripts per million
WGCNA: Weighted gene coexpression network analysis

## References

[1] Raimondi PT, Morse ANC (2000). The consequences of complex larval behavior in a coral. Ecology..

[2] Müller WA, Leitz T (2002). Metamorphosis in the cnidaria. Can J Zool NRC Res Press.

[3] Doropoulos C, Roff G, Bozec Y-M, Zupan M, Werminghausen J, Mumby PJ (2016). Characterizing the ecological trade-offs throughout the early ontogeny of coral recruitment. Ecol Monogr.

[4] Tebben J, Motti CA, Siboni N, Tapiolas DM, Negri AP, Schupp PJ, et al. Chemical mediation of coral larval settlement by crustose coralline algae. Sci Rep. 2015;5(1):10803. 10.1038/srep10803. 10.1038/srep10803 PMC4650656 26042834

[5] Tebben J, Tapiolas DM, Motti CA, Abrego D, Negri AP, Blackall LL, et al. Induction of larval metamorphosis of the coral *Acropora millepora* by tetrabromopyrrole isolated from a *Pseudoalteromonas* Bacterium. PLoS One. 2011;6(4):e19082. 10.1371/journal.pone.0019082. 10.1371/journal.pone.0019082 PMC3084748 21559509

[6] Siboni N, Abrego D, Seneca F, Motti CA, Andreakis N, Tebben J, et al. Using bacterial extract along with differential gene expression in *Acropora millepora* larvae to decouple the processes of attachment and metamorphosis. PLoS One. 2012;7:e37774. 10.1371/journal.pone.0037774. 10.1371/journal.pone.0037774 PMC3359992 22655067

[7] Sneed JM, Sharp KH, Ritchie KB, Paul VJ. The chemical cue tetrabromopyrrole from a biofilm bacterium induces settlement of multiple caribbean corals. Proc R Soc B Biol Sci. 2014;281:20133086. 10.1098/rspb.2013.3086. 10.1098/rspb.2013.3086 PMC4046396 24850918

[8] Heyward AJ, Negri AP (1999). Natural inducers for coral larval metamorphosis. Coral Reefs.

[9] Jorissen H, Galand PE, Bonnard I, Meiling S, Raviglione D, Meistertzheim A-L, et al. Coral larval settlement preferences linked to crustose coralline algae with distinct chemical and microbial signatures. Sci Rep. 2021;11(1):14610. 10.1038/s41598-021-94096-6. 10.1038/s41598-021-94096-6 PMC8285400 34272460

[10] Negri AP, Webster NS, Hill RT, Heyward AJ (2001). Metamorphosis of broadcast spawning corals in response to bacteria isolated from crustose algae. Mar Ecol Prog Ser.

[11] Hayward DC, Catmull J, Reece-Hoyes JS, Berghammer H, Dodd H, Hann SJ, et al. Gene structure and larval expression of cnox-2Am from the coral *Acropora millepora*. Dev Genes Evol. 2001;211(1):10–9. 10.1007/s004270000112. 10.1007/s004270000112 11277400

[12] Iwao K, Fujisawa T, Hatta M. A cnidarian neuropeptide of the GLWamide family induces metamorphosis of reef-building corals in the genus *Acropora*. Coral Reefs. 2002;21(2):127–9. 10.1007/s00338-002-0219-8.

[13] Erwin PM, Szmant AM (2010). Settlement induction of *Acropora* palmata planulae by a GLW-amide neuropeptide. Coral Reefs.

[14] Hatta M, Iwao K (2002). Metamorphosis induction and its possible application to coral seedlings production. Recent Adv Mar Sci Technol.

[15] Attenborough RMF, Hayward DC, Wiedemann U, Forêt S, Miller DJ, Ball EE. Expression of the neuropeptides RFamide and LWamide during development of the coral *Acropora millepora* in relation to settlement and metamorphosis. Dev Biol. 2019;446(1):56–67. 10.1016/j.ydbio.2018.11.022. 10.1016/j.ydbio.2018.11.022 30521809

[16] Takahashi T, Muneoka Y, Lohmann J, de Haro MSL, Solleder G, Bosch TCG, et al. Systematic isolation of peptide signal molecules regulating development in hydra: LWamide and PW families. Proc Natl Acad Sci. 1997;94(4):1241–6. 10.1073/pnas.94.4.1241. 10.1073/pnas.94.4.1241 PMC19775 9037037

[17] Leitz T, Morand K, Mann M. Metamorphosin A: A novel peptide controlling development of the lower metazoan *Hydractinia echinata* (Coelenterata, Hydrozoa). Dev Biol. 1994;163(2):440–6. 10.1006/dbio.1994.1160. 10.1006/dbio.1994.1160 7911112

[18] Meyer E, Aglyamova GV, Matz MV (2011). Profiling gene expression responses of coral larvae (*Acropora millepora*) to elevated temperature and settlement inducers using a novel RNA-Seq procedure. Mol Ecol.

[19] Grasso LC, Negri AP, Fôret S, Saint R, Hayward DC, Miller DJ, et al. The biology of coral metamorphosis: molecular responses of larvae to inducers of settlement and metamorphosis. Dev Biol. 2011;353(2):411–9. 10.1016/j.ydbio.2011.02.010. 10.1016/j.ydbio.2011.02.010 21338599

[20] Grasso LC, Maindonald J, Rudd S, Hayward DC, Saint R, Miller DJ, et al. Microarray analysis identifies candidate genes for key roles in coral development. BMC Genomics. 2008;9(1):540. 10.1186/1471-2164-9-540. 10.1186/1471-2164-9-540 PMC2629781 19014561

[21] Strader ME, Aglyamova GV, Matz MV (2018). Molecular characterization of larval development from fertilization to metamorphosis in a reef-building coral. BMC Genomics.

[22] Hayward DC, Hetherington S, Behm CA, Grasso LC, Forêt S, Miller DJ, et al. Differential gene expression at coral settlement and metamorphosis - A subtractive hybridization study. PLoS One. 2011;6:e26411. 10.1371/journal.pone.0026411. 10.1371/journal.pone.0026411 PMC3204972 22065994

[23] Harii S, Nadaoka K, Yamamoto M, Iwao K (2007). Temporal changes in settlement, lipid content and lipid composition of larvae of the spawning hermatypic coral *Acropora tenuis*. Mar Ecol Prog Ser.

[24] Wolfowicz I, Baumgarten S, Voss PA, Hambleton EA, Voolstra CR, Hatta M, et al. *Aiptasia* sp. larvae as a model to reveal mechanisms of symbiont selection in cnidarians. Sci Rep. 2016;6:32366. 10.1038/srep32366. 10.1038/srep32366 PMC5007887 27582179

[25] Cooke I, Ying H, Forêt S, Bongaerts P, Strugnell JM, Simakov O, et al. Genomic signatures in the coral holobiont reveal host adaptations driven by Holocene climate change and reef specific symbionts. Sci Adv. 2020;6(48):eabc6318. 10.1126/sciadv.abc6318. 10.1126/sciadv.abc6318 PMC7695477 33246955

[26] Dobin A, Davis CA, Schlesinger F, Drenkow J, Zaleski C, Jha S, et al. STAR: ultrafast universal RNA-seq aligner. Bioinformatics. 2013;29(1):15–21. 10.1093/bioinformatics/bts635. 10.1093/bioinformatics/bts635 PMC3530905 23104886

[27] Li B, Dewey CN (2011). RSEM: accurate transcript quantification from RNA-Seq data with or without a reference genome. BMC Bioinformatics..

[28] Robinson MD, McCarthy DJ, Smyth GK (2010). edgeR: a Bioconductor package for differential expression analysis of digital gene expression data. Bioinforma Oxf Engl..

[29] Jones P, Binns D, Chang H-Y, Fraser M, Li W, McAnulla C, et al. InterProScan 5: genome-scale protein function classification. Bioinforma Oxf Engl. 2014;30(9):1236–40. 10.1093/bioinformatics/btu031. 10.1093/bioinformatics/btu031 PMC3998142 24451626

[30] Young MD, Wakefield MJ, Smyth GK, Oshlack A (2010). Gene ontology analysis for RNA-seq: accounting for selection bias. Genome Biol.

[31] Moriya Y, Itoh M, Okuda S, Yoshizawa AC, Kanehisa M (2007). KAAS: an automatic genome annotation and pathway reconstruction server. Nucleic Acids Res.

[32] Kanehisa M, Sato Y (2020). KEGG mapper for inferring cellular functions from protein sequences. Protein Sci Publ Protein Soc.

[33] Liao Y, Wang J, Jaehnig EJ, Shi Z, Zhang B (2019). WebGestalt 2019: gene set analysis toolkit with revamped UIs and APIs. Nucleic Acids Res.

[34] Mistry J, Chuguransky S, Williams L, Qureshi M, Salazar GA, Sonnhammer ELL, et al. Pfam: the protein families database in 2021. Nucleic Acids Res. 2021;49(D1):D412–9. 10.1093/nar/gkaa913. 10.1093/nar/gkaa913 PMC7779014 33125078

[35] Frickey T, Lupas A (2004). CLANS: a Java application for visualizing protein families based on pairwise similarity. Bioinformatics..

[36] Putnam NH, Srivastava M, Hellsten U, Dirks B, Chapman J, Salamov A, et al. Sea anemone genome reveals ancestral eumetazoan gene repertoire and genomic organization. Science. 2007;317(5834):86–94. 10.1126/science.1139158. 10.1126/science.1139158 17615350

[37] Bauknecht P, Jékely G (2015). Large-scale combinatorial deorphanization of Platynereis neuropeptide GPCRs. Cell Rep.

[38] Protein Sequence Analysis Using the MPI Bioinformatics Toolkit. Gabler F, Nam SZ, Till S, Mirdita M, Steinegger M, Söding J, Lupas AN, Alva V. Curr Protoc Bioinformatics. 2020;72(1):e108. 10.1002/cpbi.108. 10.1002/cpbi.108 33315308

[39] Langfelder P, Horvath S (2008). WGCNA: an R package for weighted correlation network analysis. BMC Bioinformatics.

[40] Wickham H (2016). ggplot2: Elegant graphics for data analysis.

[41] Reyes-Bermudez A, Lin Z, Hayward DC, Miller DJ, Ball EE. Differential expression of three galaxin-related genes during settlement and metamorphosis in the scleractinian coral *Acropora millepora*. BMC Evol Biol. 2009;9(1):178. 10.1186/1471-2148-9-178. 10.1186/1471-2148-9-178 PMC2726143 19638240

[42] Hirose M, Yamamoto H, Nonaka M. Metamorphosis and acquisition of symbiotic algae in planula larvae and primary polyps of *Acropora* spp. Coral Reefs. 2008;27(2):247–54. 10.1007/s00338-007-0330-y.

[43] Haryanti D, Hidaka M (2019). Developmental changes in the intensity and distribution pattern of green fluorescence in coral larvae and juveniles. Galaxea J Coral Reef Stud.

[44] Aihara Y, Maruyama S, Baird AH, Iguchi A, Takahashi S, Minagawa J (2019). Green fluorescence from cnidarian hosts attracts symbiotic algae. Proc Natl Acad Sci.

[45] Rachdi L, Maugein A, Pechberty S, Armanet M, Hamroune J, Ravassard P, et al. Regulated expression and function of the GABA_B_ receptor in human pancreatic beta cell line and islets. Sci Rep. 2020;10(1):13469. 10.1038/s41598-020-69758-6. 10.1038/s41598-020-69758-6 PMC7417582 32778664

[46] Hobmayer B, Rentzsch F, Kuhn K, Happel CM, von Laue CC, Snyder P, et al. WNT signalling molecules act in axis formation in the diploblastic metazoan Hydra. Nature. 2000;407(6801):186–9. 10.1038/35025063. 10.1038/35025063 11001056

[47] Plickert G, Jacoby V, Frank U, Müller WA, Mokady O (2006). Wnt signaling in hydroid development: formation of the primary body axis in embryogenesis and its subsequent patterning. Dev Biol.

[48] Konovalova A, Søgaard-Andersen L, Kroos L (2014). Regulated proteolysis in bacterial development. FEMS Microbiol Rev.

[49] Lecointe A, Domart-Coulon I, Paris A, Meibom A (2016). Cell proliferation and migration during early development of a symbiotic scleractinian coral. Proc R Soc B Biol Sci.

[50] Cleves PA, Strader ME, Bay LK, Pringle JR, Matz MV (2018). CRISPR/Cas9-mediated genome editing in a reef-building coral. Proc Natl Acad Sci.

