# Supplementary figures and images for: Gene expression alterations from reversible to irreversible stages during coral metamorphosis
Source: Zoological Lett. 2022 Jan 25;8:4. doi: 10.1186/s40851-022-00187-1 (PMC8787945; doi:10.1186/s40851-022-00187-1)

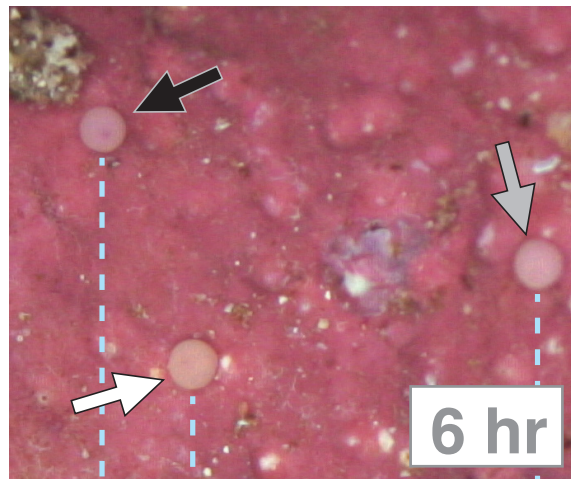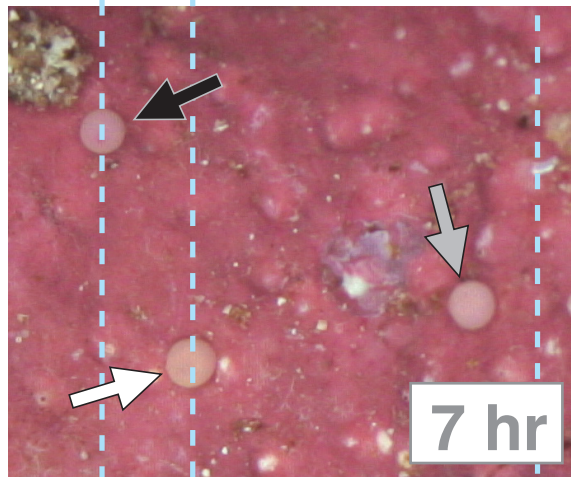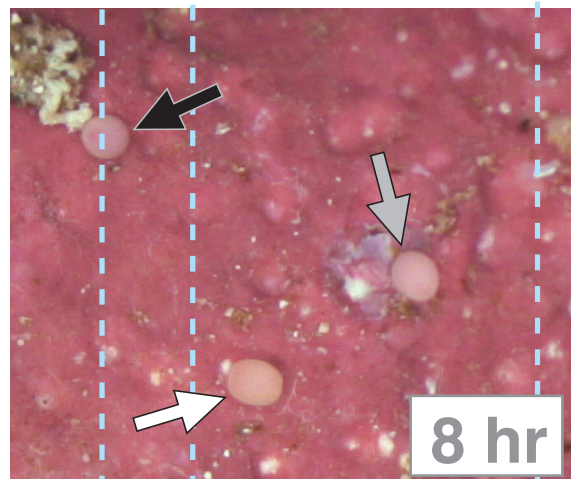

Supplement: Supplementary file 1 — Additional file 1: Supplementary Fig. 1. Planula larvae returning to the motile form after resting on the substrata. Acropora tenuis planula larvae were released on rubble covered by CCA. Pictures taken 6, 7, and 8 h after release are shown (A, B, and C, respectively). Dotted lines indicate the positions of the larvae at 6 h. [file 40851_2022_187_MOESM1_ESM.pdf]
